# Rate control deficits during pinch grip and ankle dorsiflexion in early-stage Parkinson’s disease

**DOI:** 10.1371/journal.pone.0282203

**Published:** 2023-03-03

**Authors:** Jae Woo Chung, Christopher A. Knight, Abigail E. Bower, Justin P. Martello, John J. Jeka, Roxana G. Burciu

**Affiliations:** 1 Department of Kinesiology and Applied Physiology, University of Delaware, Newark, DE, United States of America; 2 Interdisciplinary Neuroscience Graduate Program, University of Delaware, Newark, DE, United States of America; 3 Department of Neurosciences, Christiana Care Health System, Newark, DE, United States of America; University of Catania, ITALY

## Abstract

**Background:**

Much of our understanding of the deficits in force control in Parkinson’s disease (PD) relies on findings in the upper extremity. Currently, there is a paucity of data pertaining to the effect of PD on lower limb force control.

**Objective:**

The purpose of this study was to concurrently evaluate upper- and lower-limb force control in early-stage PD and a group of age- and gender-matched healthy controls.

**Methods:**

Twenty individuals with PD and twenty-one healthy older adults participated in this study. Participants performed two visually guided, submaximal (15% of maximum voluntary contractions) isometric force tasks: a pinch grip task and an ankle dorsiflexion task. PD were tested on their more affected side and after overnight withdrawal from antiparkinsonian medication. The tested side in controls was randomized. Differences in force control capacity were assessed by manipulating speed-based and variability-based task parameters.

**Results:**

Compared with controls, PD demonstrated slower rates of force development and force relaxation during the foot task, and a slower rate of relaxation during the hand task. Force variability was similar across groups but greater in the foot than in the hand in both PD and controls. Lower limb rate control deficits were greater in PD with more severe symptoms based on the Hoehn and Yahr stage.

**Conclusions:**

Together, these results provide quantitative evidence of an impaired capacity in PD to produce submaximal and rapid force across multiple effectors. Moreover, results suggest that force control deficits in the lower limb may become more severe with disease progression.

## Introduction

Parkinson’s disease (PD) is a complex, progressive degenerative disorder of the central nervous system that affects the way people move [1–3]. Currently accepted diagnostic criteria require the presence of bradykinesia and at least one other motor symptom such as rest tremor, rigidity, or postural instability [4–8]. Bradykinesia, one of the early symptoms of PD, is also known as hypokinesia, and typically emerges when dopamine levels in the brain decline because of a progressive loss of dopamine-producing neurons in the substantia nigra pars compacta and the appearance of intracellular inclusions, also known as Lewy bodies [8–12]. It is estimated that by the time the first motor symptoms are noticed, there is about 50–60% loss of dopaminergic neurons in the substantia nigra pars compacta and up to 80% loss of dopaminergic terminals, particularly in the putamen [13–15].

Bradykinesia manifests as a slowing of movements, with a decrease in amplitude and speed as the movements continue [16, 17]. It affects multiple phases of movement including movement preparation, movement initiation and execution [18, 19], and can impact an individual’s ability to carry out activities of daily living [20, 21]. Bradykinesia is common in the upper extremity, causing difficulties with basic movements involving the hands such as buttoning a shirt, cutting food, tying shoelaces. An individual may also present with a reduction in automatic movements such as reduced swinging of the arms when walking [22–24]. It is important to note however that bradykinesia can affect more than one limb. It can affect one side of the body or the whole body. The extent of motor disability is related to the progressive nature of PD. Symptoms start in the upper extremity and progress to affect the lower extremity [25]. With time, PD symptoms worsen [26]. Bradykinesia will eventually cause a person to drag the feet when walking, take shorter steps and overall walk slowly [27].

Thus far, numerous studies have explored changes within the neuromuscular system underlying motor symptoms in PD (including bradykinesia), using various motor tasks: hand joystick movements [28], arm abduction [29], elbow flexion [30–36], hand grip and precision grip force production tasks [37–44], repeated index finger tapping [45, 46], wrist flexion and extension tasks [47, 48], ankle plantarflexion and dorsiflexion tasks, and hip muscles force tasks [49, 50]. Of note, many of these prior investigations focused on upper limb function. Specifically, force control deficits in the upper limb in PD are well-characterized by the motor control literature. Isometric force control paradigms are commonly used as they allow the study of the generation and relaxation of force, neural processes regulated to a great extent by dopamine release, and affected by PD [39]. A common observation is that individuals with PD exhibit a slower rate of force development [36, 37, 42, 44, 51–55] and force relaxation [35, 37, 40, 42, 52, 56, 57] during grip and pinch force tasks. Lower limb force control studies in PD are scarce compared to upper limb studies [32, 38–41, 58], and have focused primarily on lower limb muscle strength and force variability [50]. Lower limb bradykinesia in PD is understudied and represents an important limiting factor of the quality of life in this clinical population. A thorough characterization of force control deficits in the lower limb in PD is essential since lower extremity deficits are prevalent in the PD community [19]. Of great importance is also determining the severity of these deficits in the early stages of PD. Such deficits could be subtle in the early stages of the disease (i.e., Hoehn and Yahr stages 1–2) and challenging to document through a standard clinical examination such as the Movement Disorder Society Sponsored Revision of the Unified Parkinson’s Disease Rating Scale (MDS-UPDRS) [59]. While MDS-UPDRS represents the gold standard diagnostic tool in PD and its motor section (part III) is used to assess the severity and progression of motor symptoms in PD [8, 60], it is important to note that only two items included in this clinical scale are used to assess the severity of bradykinesia in the lower extremity (items 3.7—toe tapping, and 3.8—leg agility). Therefore, a better understanding of lower limb impairment in PD and a more objective, instrumented evaluation of motor deficits in PD is necessary and crucial to enhancing the design of rehabilitative interventions across the different stages of the disease.

Given the importance of adequate force control of the lower limbs for mobility, our purpose was to extend the PD literature by 1) evaluate force control during a submaximal, isometric lower limb task in a group of early-stage PD and a group of healthy older adults, and 2) compare for the first time upper and lower force control deficits in the same cohort of PD patients. We hypothesized that compared to the control group, PD would exhibit a reduction in the rates of force development and force relaxation in both the upper and lower limbs and would have increased force variability in the lower limb compared to the upper limb. Finally, we expected force control deficits in the lower limb as detected by quantitative measures to be greater than upper limb deficits, a pattern likely not mirrored by the clinical assessment which is known to provide an incomplete and coarse evaluation of lower limb symptoms.

## Methods

### Participants

This study included 41 participants: 20 individuals with early-stage idiopathic PD, without dementia or concurrent movement disorders (mean age = 67.30 ± 8.78, 11 males), and 21 older adults with no known neurological or musculoskeletal disorders (mean age: 64.05 ± 7.97, 7 males). Participants with PD were diagnosed by a movement disorder specialist using the United Kingdom PD Brain Bank Diagnostic Criteria [7] and were recruited either from the Christiana Care Neurology Specialists Clinic in Newark, Delaware, or the local PD community. Most of the healthy participants were recruited by advertisements in the Newark area and a few were caregivers of the PD participants (non-blood relatives). Importantly, the PD and control groups were matched for age, sex, cognitive status, handedness, and tested side. The demographic and clinical characteristics of the two groups are listed in Table 1. All study-related assessments took place in the morning, at the University of Delaware. The PD group was tested following an overnight withdrawal from antiparkinsonian medication (approximately 12 to 14 hours after the last dose of PD medication). The study was approved by the University of Delaware Institutional Review Board and performed in accordance with the guidelines established in the Declaration of Helsinki. All procedures were carried out with the adequate understanding and written consent of the participants involved in the research.

**Table 1:** 

Variables	Healthy Controls	Parkinson’s Disease	P- Value
N	21	20	n/a
Age (Years)	64.05 (7.97)	67.30 (8.78)	0.223
Sex (Male | Female)	7 | 14	11 | 9	0.162
Handedness (Right | Left)	19 | 2	18 |2	0.959
Tested Side (Right | Left)	13 | 8	7 | 13	0.085
Tested Side (Dominant | Non-Dominant)	11 | 10	9 |11	0.636
MVC–Tested Hand (N)	52.10 (20.01)	49.75 (19.76)	0.708
MVC–Tested Foot (N)	71.48 (44.49)	54.05 (40.98)	0.199
MoCA	27.67 (2.20)	26.95 (2.28)	0.313
H & Y	n/a	1.85 (0.59)	n/a
Disease Duration (Months)	n/a	56.85 (44.03)	n/a
More Affected Side (Right | Left)	n/a	7 | 13	n/a
Total LEDD	n/a	530.40 (589.32)	n/a
MDS-UPDRS-III–Total Score	2.29 (3.13)	31.20 (10.77)	<0.001
MDS-UPDRS-III–Tested Hand	0.90 (1.30)	18.50(18.79)	<0.001
MDS-UPDRS-III–Other Hand	1.10 (1.41)	7.75 (6.63)	<0.001
MDS-UPDRS-III–Tested Foot	0.52 (0.87)	5.30 (3.85)	<0.001
MDS-UPDRS-III–Other Foot	0.52 (0.87)	5.00 (4.88)	<0.001
MDS-UPDRS-III–Bradykinesia Tested Hand	0.76 (1.14)	8.90 (4.18)	<0.001
MDS-UPDRS-III–Bradykinesia Other Hand	1.00 (1.38)	4.50 (2.28)	<0.001
MDS-UPDRS-III–Bradykinesia Tested Foot	0.24 (0.54)	3.40 (1.54)	<0.001
MDS-UPDRS-III–Bradykinesia Other Foot	0.14 (0.36)	2.40 (2.46)	<0.001
Kinesia ONE–Total Score	n/a	20.28 (5.82)	n/a
Kinesia ONE–Tested Side–Finger Taps Speed	n/a	1.60 (0.90)	n/a
Kinesia ONE–Tested Side–Finger Taps Amplitude	n/a	2.34 (0.90)	n/a
Kinesia ONE–Tested Side–Finger Taps Rhythm	n/a	0.96 (0.54)	n/a
Kinesia ONE–Tested Side–Hand Movement Speed	n/a	1.60 (0.60)	n/a
Kinesia ONE–Tested Side–Hand Movement Amplitude	n/a	1.72 (0.76)	n/a
Kinesia ONE–Tested Side–Hand Movement Rhythm	n/a	0.86 (0.90)	n/a
Kinesia ONE–Tested Side–Rapid Alternating Movement Speed	n/a	1.77 (0.62)	n/a
Kinesia ONE–Tested Side–Rapid Alternating Movement Amplitude	n/a	1.43 (0.85)	n/a
Kinesia ONE–Tested Side–Rapid Alternating Movement Rhythm	n/a	0.81 (0.95)	n/a
Kinesia ONE–Tested Side–Toe Taps	n/a	2.00 (0.64)	n/a
Kinesia ONE–Tested Side–Leg Lifts	n/a	1.28 (0.50)	n/a

The table lists the sociodemographics, clinical characteristics and several behavioral measures for the two groups. The last column lists the *p*-values of the statistical analyses described in the “Statistical Analysis” section. Data represent the count or mean ± 1 standard deviation (SD). Of note, all tests were completed by the PD patients in the “off” state, following an overnight withdrawal from medication. Disease duration is defined as time since diagnosis. Kinesia ONE scores for the tested side are reported. The Kinesia ONE total score is the sum of all items (left + right side items listed in the table plus scores for rest tremor, postural tremor, kinetic tremor, gait, and dyskinesia). Abbreviations: H & Y = Hoehn and Yahr Staging Scale; LEDD = levodopa equivalent daily dose; MoCA = Montreal Cognitive Assessment; MDS-UPDRS-III = the motor section of the Movement Disorder Society Unified Parkinson’s Disease Rating Scale; mg = milligram; MVC = maximum voluntary contraction; N = Newtons.

### Force data acquisition

**Fig 1:**
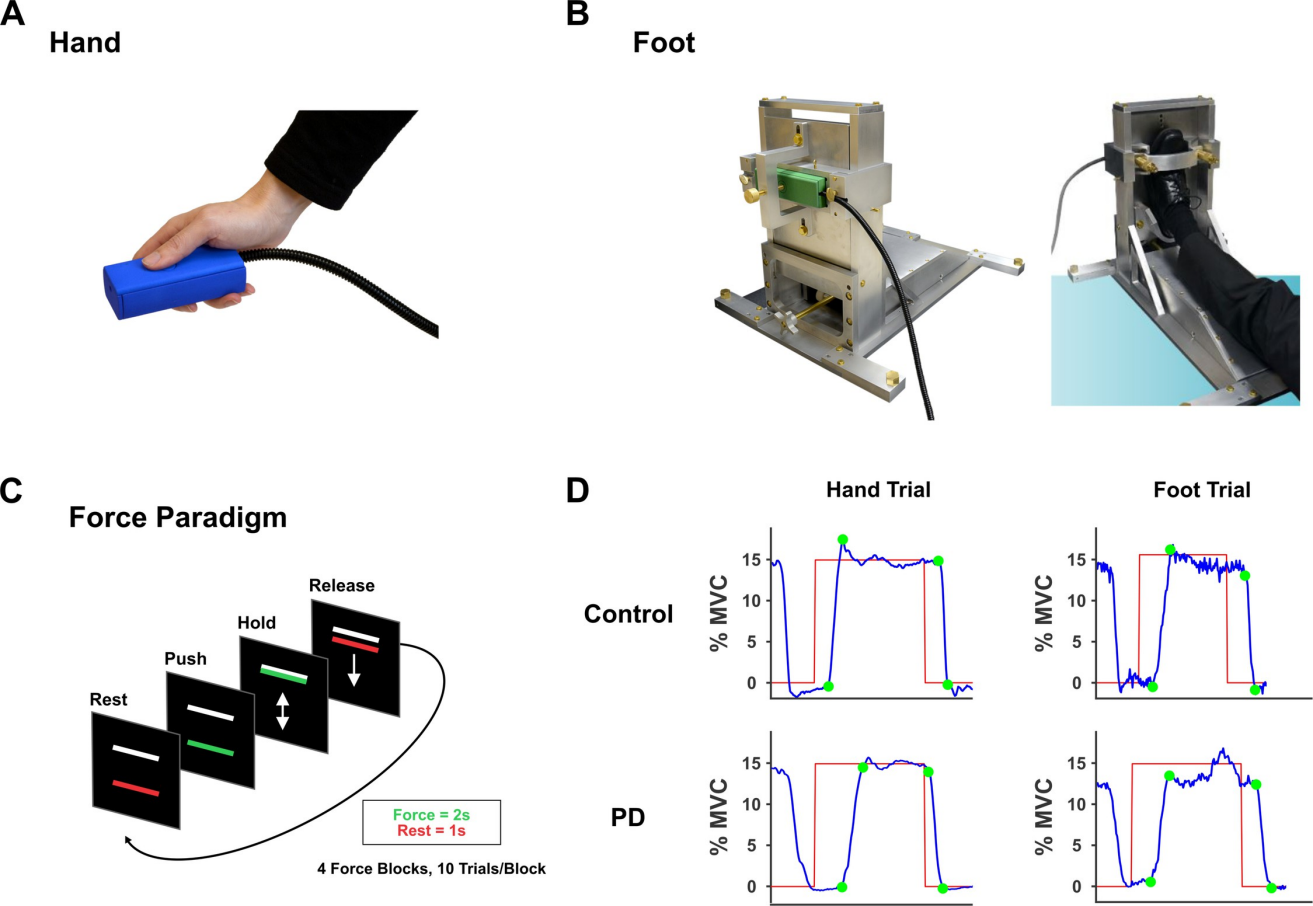
Experimental setup and paradigm. (A) Hand force device that measured pinch force. (B) Custom-designed foot device that measured the force produced by dorsiflexing the ankle. (C) Depiction of the force paradigm. After a 30s rest period, the red force bar turned green to cue force generation. The participants had to produce force for 2s and relax for 1s (1 trial = 3s, 10 trials per block/cycle). Instructions were to produce force as quick as possible and match the white target bar. When the bar turned red again, the participants rested for 1s. There were 4 30-s blocks of force production, and each force block was followed by a 30-s resting period. (D) Sample force data illustrating a trial per task for one representative control and one PD. For each force trial, four points were defined: (*p1*) onset of force, (*p2*) onset of the steady period during which the amplitude of the force bar matched the amplitude of the target bar, (*p3*) offset of the steady period, and (*p4*) offset of force.

Two force production tasks were performed by each participant: (1) a hand task, and (2) a foot task. During the two tasks, participants produced force against custom-designed Bragg grating fiber-optic force transducers (Neuroimaging Solutions, Gainesville, FL) (Fig 1A and 1B). The sensors were housed in a 3D printed apparatus to insulate them from heat. Force signals were transmitted through a fiber-optic cable to a SI155 Micron Hyperion Optical Sensing Interrogator (Micron Optics, Atlanta, Georgia). Force data was digitized at 50 Hz using the interrogator and converted to Newtons (N) with custom data collection software written in LabVIEW (National Instruments, Austin, TX). Online visual feedback of the force output was visible to participants on a 32” 1920 x 1080 widescreen LCD display at 120 Hz refresh rate. Force data were low-pass filtered using a Butterworth, 20 Hz 4^th^-order dual-pass filter.

### Force paradigm and experimental procedures

PD participants were tested on their more affected side, whereas control participants were tested either on the dominant or non-dominant side (side was randomized; see Table 1). For the foot task, a custom-designed foot force device was used that supported the participant’s tested foot in a comfortable position (Fig 1). The foot was placed in an adjustable, secure strap that allowed isometric force generation. The strap secured the foot at the level of the metatarsals. Force was generated by ankle dorsiflexion and measured by force sensor that could measure up to 700N. Each time a participant dorsiflexed the ankle, a piston at the back of the foot device applied compressive force to the force sensor. For the hand task, a force sensor which could measure up to 150N was used. The sensor was held between the thumb and index finger in a modified precision grip (Fig 1). Each participant’s maximum voluntary contraction (MVC) was measured prior to beginning data collection. Participants were able to see a horizontal bar on the displayed on a black background on the computer screen. A change in color of this bar from red to green instructed participants to produce maximum force for 5s during 3 consecutive trials (5s rest period between trials). The peak force during these 3 trials was used to calculate an average MVC per task, which would then be used to normalize force demands across participants. During the experiment, force data were collected while each participant produced force to a target level set at 15% of their MVC for each effector (hand/foot) separately. The order of the hand and foot tasks was randomized. Importantly, all participants completed a 10-15-minute training session prior to beginning the data collection to familiarize themselves with the force tasks and minimize learning effects during the experiment. A representation of the force paradigms is shown in Fig 1C. Each task involved nine 30-second blocks. The tasks began with a 30-second rest block followed by four cycles of 30s of force alternating with 30s of rest. While performing the force tasks, participants were provided with online visual feedback about their task performance in the form of a moving bar. During the force blocks, two horizontal bars were displayed on a black background, on the computer screen. The top bar was white and represented the target (set for each participant and limb at 15% of MVC), whereas the bottom bar was colored and represented the participant’s force. This force bar would move vertically on the screen depending on the amount of force applied to the sensors. Each block consisted of 10 trials (3s each: 2s force, 1s rest). Participants were instructed to generate force for 2s and relax for 1s. Instructions were to be quick in generating the force and produce enough force to bring the colored bar on top of the white bar. Therefore, instructions emphasized both speed and accuracy.

### Motor and Cognitive Assessment

The presence and severity of motor symptoms along with the cognitive status were assessed in both groups using the motor section of MDS-UPDRS-III and the Montreal Cognitive Assessment (MoCA), respectively [61, 62]. To quantify the severity of specific manifestations of PD, several subscores were calculated based on subsets of MDS-UPDRS-III items: MDS-UPDRS-III tested hand/other hand, MDS-UPDRS-III tested foot/other foot, MDS-UPDRS-III bradykinesia tested hand/other hand, and MDS-UPDRS-III bradykinesia tested foot/other foot. The PD group also underwent an additional instrumented assessment of motor symptoms using the Kinesia ONE wearable sensor (Great Lakes NeuroTechnologies Inc, Cleveland, OH). The Kinesia ONE device is approved by the Food and Drug Administration (FDA), provides clinically validated objective outcomes to track the severity of motor symptoms, and is used around the globe in clinical trials for PD, essential tremor, and other movement disorders [63–65]. The sensor includes a triaxial accelerometer and gyroscope and is designed to be worn on the index finger or shoe heel depending on the symptom being assessed. In this study, both sides were tested using pre-defined tasks based on the MDS-UPDRS-III scale. Data from the motion sensor was used to calculate severity scores on a 0–4 rating scale for specific upper limb tasks available in the MDS-UPDRS-III such as finger tapping, hand movements, pronation-supination movements of hands, postural tremor of the hands, kinetic tremor of the hands, rest tremor of the hands (i.e., MDS-UPDRS-III 3.4, 3.5, 3.6, 3.15, 3.16, and 3.17 respectively), lower limb tasks such as toe tapping, leg agility, gait (i.e., MDS-UPDRS-III 3.7, 3.8, 3.10 and 3.11 respectively), and general symptoms such as dyskinesia. Unlike the MDS-UPDRS-III, Kinesia ONE also provides severity scores for key movement characteristics such as speed, amplitude, and rhythm for all upper limb tasks measuring bradykinesia. The bradykinesia-related scores for the tested side are listed in Table 1. Importantly, all motor tests in PD were conducted off antiparkinsonian medication [66], with testing following an overnight withdrawal from PD medication of at least 12 hours.

### Force data analysis

Force data collected during the hand and foot tasks were analyzed using custom algorithms in MATLAB R2021b (The Mathworks, Natick, MA). First, data were filtered using a 6th-order Butterworth filter with a cutoff frequency of 15 Hz. For each force trial, four points were defined consistent with previous analyses: (*p1*) onset of force, (*p2*) onset of the steady period during which the amplitude of the force bar matched the amplitude of the target bar, (*p3*) offset of the steady period, and (*p4*) offset of force [37]. Fig 1D illustrates sample force data from one control and one PD along with these markings. The outcome variables described below were calculated based on the following phases of force production: a) force increase (*p1—p2*), b) steady force (*p2—p3*), and c) force decrease (*p3 –p4*). Importantly, all force measures were calculated using the filtered force data and for each trial. The force measures subjected to statistical analysis represent the average for all force trials for each task and participant. First, we calculated the force amplitude during the steady period (normalized by MVC) and trial duration (s; time between *p1* and *p4*). Then, the following speed-related measures which would be indicators of bradykinesia were calculated: normalized rate of force increase (i.e., the rate of force increase normalized by MVC; % MVC/s) and normalized rate of force decrease (i.e., the rate of force decrease normalized by MVC; % MVC/s). In addition to speed-related measures, we also calculated several variability-related measures to assess differences in the accuracy of the task performance: the standard deviation (SD) of the normalized force amplitude (% MVC), the constant force error (N), and absolute force error (N).

### Statistical analysis

All statistical analyses were performed in SPSS 28.0 (IBM, New York). First, a visual inspection of the data, using histograms and Q-Q plots, was performed. Next, outcome measures were assessed for normality and equal variance with the Saphiro-Wilk and Levene’s Tests. The results to these tests motivated the choice for parametric or non-parametric testing. Categorical data (i.e., sex, handedness, tested side based on body side or dominance) were compared between groups using a Chi-Square test. All the continuous data (see Table 1), except for the force data and Kinesia ONE scores, were compared between groups using an Independent Samples T-Test. Since the force data did not meet the assumption for normality (*p-values* for the Saphiro-Wilk Test < 0.05), we proceeded with non-parametric statistics. Specifically, for each task the Mann-Whitney U Test was used to assess group differences in the following measures: normalized force amplitude, normalized rate of force increase, normalized rate of force decrease, SD of the normalized force amplitude, constant force error, absolute force error, and trial duration. For the same force measures, we performed a Wilcoxon Signed-Rank Test to assess limb differences (hand vs. foot), separately for PD and controls. Significance for statistical tests was set an α = 0.05. Spearman’s Rank-Order correlation analyses were conducted in the PD group with the goal to assess the relation between force control deficits (as determined by the previous group analyses) and the severity of motor symptoms as assessed by the total MDS-UPDRS-III, MDS-UPDRS-III bradykinesia subscores corresponding to the tested hand and foot, and the Kinesia ONE scores for the tested hand and foot. Additionally, we conducted a Mann-Whitney U Test to assess differences in speed-related force measures between patients in the earlier vs. more advanced stages of the disease. For this analysis, PD were grouped as PD with a Hoehn and Yahr stage 1 (i.e., unilateral symptoms; n = 5) and PD with a Hoehn and Yahr stage ≥ 2 (bilateral symptoms; n = 15: 14 Hoehn and Yahr Stage 2 + 1 Hoehn and Yahr Stage 3). More information on the clinical characteristics of these two PD groups is available in Table 3.

## Results

### Sociodemographic, clinical and behavioral outcomes

Importantly, there were no significant group differences in age, sex, cognitive status based on the MoCA test, handedness (right vs. left), tested side (right vs. left or dominant vs. non-dominant), MVC of the tested hand and foot (*p-values* for the Independent Samples T-Tests and Chi-Square Tests > 0.05; Table 1). As expected, the MDS-UPDRS-III total score along with all the subscores presented in Table 1 were greater in the PD group compared with the control group (*p-values* < 0.001; Table 1). Of note, the MDS-UPDRS-III was used in the control group to rule out potential parkinsonian symptoms.

### Force outcomes

**Fig 2:**
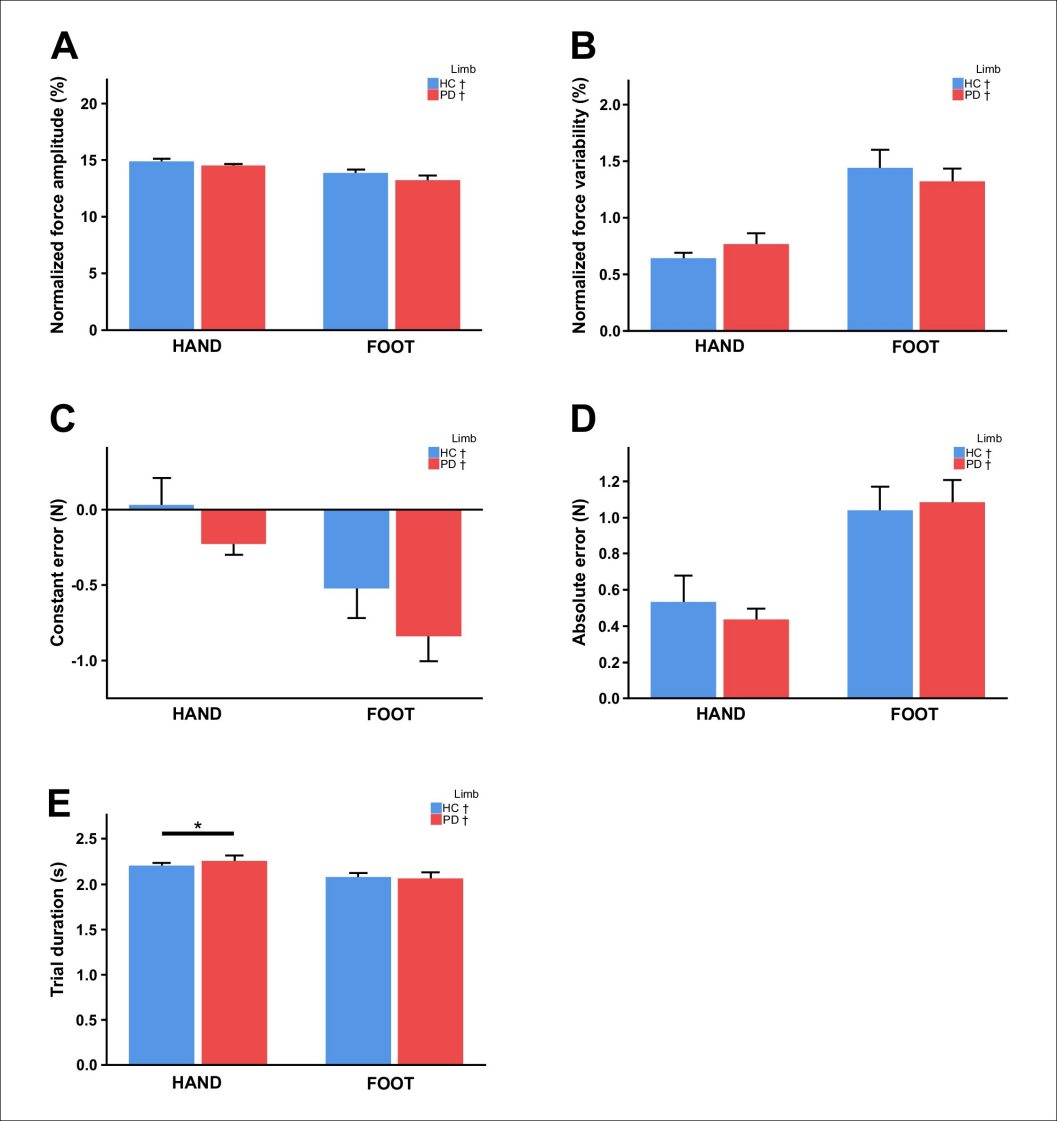
Bar graphs represent task performance for each group and limb (mean ±1 SE). (A) Normalized force amplitude, (B) SD of the normalized force amplitude, (C) constant error, (D) absolute error, (E) trial duration. Asterisks indicate significant group effects at *p* < 0.05. Obelisks indicate significant limb effects at *p* < 0.05. Abbreviations: HC = healthy controls, PD = Parkinson’s disease.

**Fig 3:**
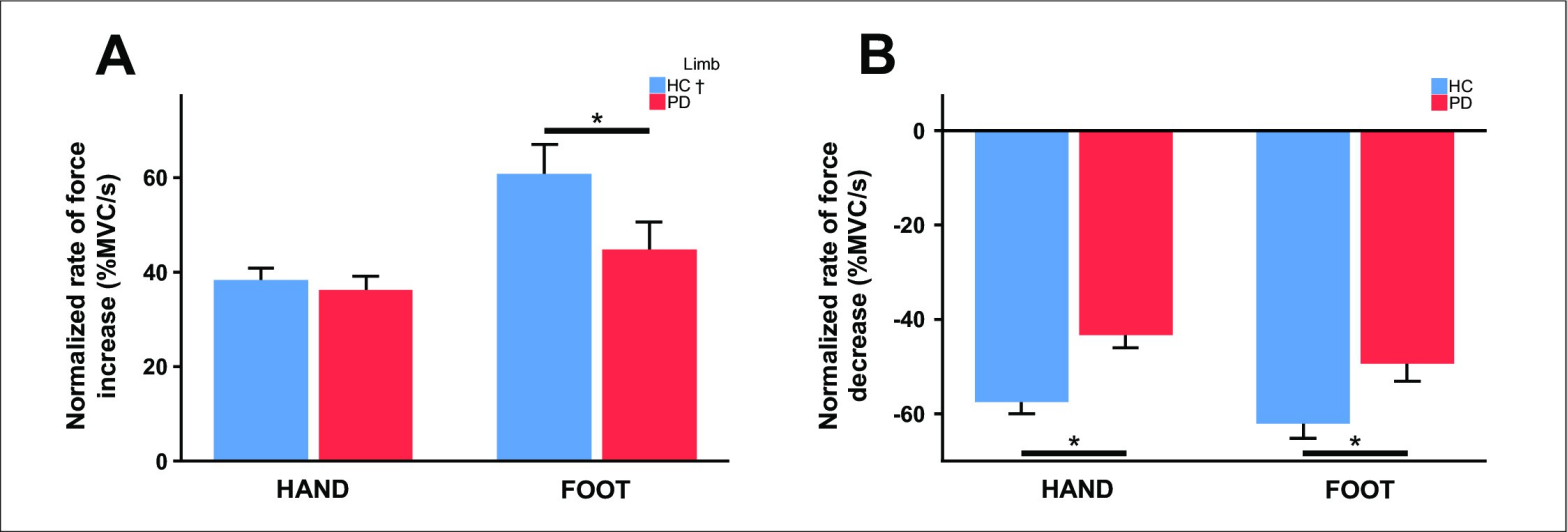
Bar graphs represent task performance for each group and limb (mean ±1 SE). (A) Normalized rate of force increase, (B) normalized rate of force decrease. Asterisks indicate significant group effects at *p* < 0.05. Obelisks indicate significant limb effects at *p* < 0.05. Abbreviations: HC = healthy controls, PD = Parkinson’s disease.

**Fig 4:**
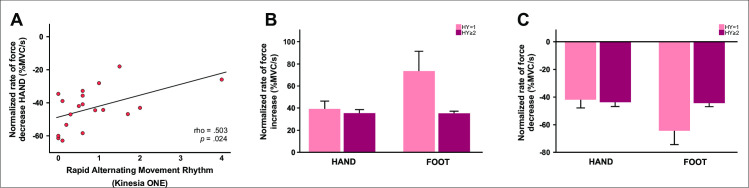
(A) A Spearman’s Rank-Order correlation analysis revealed a significant correlation in PD between the severity of rhythm impairment during the pronation-supination task from the Kinesia ONE protocol and the rate of force relaxation during the hand task. (B) Normalized rate of force increase for the hand and foot tasks broken down by Hoehn and Yahr stage. (C) Normalized rate of force decrease for the hand and foot tasks broken down by Hoehn and Yahr stage. As one can see in the bar graphs which indicate the mean ±1 SE, PD with a Hoehn and Yahr stage 1 (unilateral symptoms) were faster at generating and releasing force during the foot task than PD with a Hoehn and Yahr stage ≥ 2 (bilateral symptoms). There was no association between the progression staging system in PD and performance on the hand task. Abbreviations: HY = Hoehn and Yahr Staging Scale.

Force measures are summarized in Table 2 and presented in Figs 2 and 3. First, we found no group difference in the mean force amplitude produced during the two force tasks or any of the variability-related force measures (i.e., SD of normalized force amplitude, constant error, and absolute error) (*p-values* Mann-Whitney U Tests > 0.05, Fig 2, Table 2). That is, both groups were able to generate the required force and force accuracy was similar in the PD and control groups. The duration of the force trial was slightly longer in the PD group during the hand task (U = 129.5, *p* = 0.036, Table 2) but not during the foot task (*p* = 0.969). However, speed-related measures differed between groups. Compared to controls, PD had a lower rate of force increase and force decrease during the foot task (normalized rate of force increase _FOOT_: U = 127.0, *p* = 0.030; normalized rate of force decrease _FOOT_: U = 97.0, *p* = 0.003), and a lower rate of force decrease during the hand task (normalized rate of force decrease _HAND_: U = 85.0, *p* = 0.001) (Fig 3, Table 2). The rate of force increase during the hand task did not differ between groups (*p* = 0.754) (Fig 4, Table 2).

**Table 2:** 

Variables	HC				PD				*P*-Value			
	HAND		FOOT		HAND		FOOT		HAND	FOOT	HC	PD
	Mean	SD	Mean	SD	Mean	SD	Mean	SD	HC vs PD	HC vs PD	HAND vs FOOT	HAND vs FOOT
Normalized Force Amplitude (% MVC)	14.88	1.21	13.90	1.40	14.52	0.69	13.22	1.97	0.514	0.273	**0.010**	**0.015**
SD of Normalized Force Amplitude (% MVC)	0.65	0.21	1.44	0.73	0.77	0.42	1.32	0.50	0.584	0.676	**<0.001**	**0.003**
Constant Error (N)	0.03	0.81	-0.52	0.90	-0.23	0.32	-0.84	0.75	0.465	0.375	**0.003**	**0.007**
Absolute Error (N)	0.53	0.66	1.04	0.59	0.44	0.26	1.08	0.56	0.938	0.639	**0.001**	**<0.001**
Normalized Rate of Force Increase (% MVC/s)	38.32	11.77	60.93	28.73	36.32	13.12	44.87	25.86	0.754	**0.030**	**0.002**	0.411
Normalized Rate of Force Decrease (% MVC/s)	-57.39	12.17	-62.04	14.61	-43.24	12.13	-49.40	15.99	**0.001**	**0.003**	0.122	0.108
Trial Duration (s)	2.21	0.12	2.08	0.22	2.26	0.28	2.07	0.30	**0.036**	0.969	**0.014**	**0.004**

The table the force outcome measures derived from the hand and foot force production tasks. Data are presented for each group and limb in the form of means ± 1 standard deviation (SD). The *p*-values corresponding to the statistical analyses described statistical analyses described in the “Statistical Analysis” section are also included. Values highlighted in bold indicate statistically significant results at *p* <0.05. Abbreviations: HC = healthy controls; MVC = maximum voluntary contraction; N = Newtons; PD = Parkinson’s disease; s = second.

The limb analysis revealed a slightly greater normalized force amplitude during the hand task compared to the foot task in both PD and controls (HC: Z = -2.589, *p* = 0.010; PD: Z = -2.427, *p* = 0.015). A similar pattern was observed for the force trial duration, this being slightly longer during the hand task than during the foot task (HC: Z = -2.468, *p* = 0.014; PD: Z = -2.875, *p* = 0.004). As expected, in both groups, force variability measured by the SD of the normalized force amplitude, constant error (CE) and absolute error (AE) was greater in the foot than in the hand (HC: Z_SD_ = -3.702, *p* < 0.001; PD: Z_SD_ = -2.949, *p* = 0.003; HC: Z_CE_ = -2.937, *p* = 0.003; PD: Z_CE_ = -2.688, *p* = 0.007; HC: Z_AE_ = -3.180, *p* = 0.001; PD: Z_AE_ = -3.509, *p* < 0.001). Finally, controls were faster at generating force with the ankle than the hand (Z = -3.076, *p* = 0.002). By contrast no limb differences were in found in the PD group (*p* = 0.411). The rate of force relaxation did not differ between limbs in either group (*p-values* > 0.05).

### Relation between force measures and disease severity

In the PD group, we examined the relationship between force outcomes that differed significantly between groups (i.e., normalized rate of force decrease _HAND_, normalized rate of force increase _FOOT_, normalized rate of force decrease _FOOT_) and disease severity measured by the total MDS-UPDRS-III, MDS-UPDRS-III bradykinesia subscores for the tested hand and foot, as well as the Kinesia ONE scores for the tested hand and foot. We also contrasted force measures between PD with a Hoehn and Yahr of 1 (i.e., unilateral symptoms) and PD with a Hoehn and Yahr ≥ 2 (i.e., bilateral symptoms). The Spearman’s Rank-Order correlation analyses were limb specific such that hand-specific force measures were correlated with hand-specific clinical measures, and foot-specific force measures were correlated with foot-specific clinical measures. In the PD group, a positive correlation was found between the normalized rate of force decrease _HAND_ and the severity of rhythm impairment during the pronation-supination task from the Kinesia ONE protocol performed with the tested hand (rho = 0.503, *p* = 0.024) (Fig 4A). Therefore, PD who had a slower rate of force decrease during the hand force task, also had a higher score/more interruption in rhythm while performing rapid alternating movements with their more affected hand. Within the PD group, speed-related measures during the foot task differed based on the severity of disease as assessed by the Hoehn and Yahr stage. PD in the earlier stages of the disease had greater force development and force relaxation during the ankle dorsiflexion task than PD in the later stages of the disease (normalized rate of force increase _FOOT_: U = 4.0, *p* = 0.003; normalized rate of force decrease _FOOT_: U = 13.0, *p* = 0.032) (Fig 4B and 4C). However, the rate of force increase and decrease during the hand task did not differ between groups (*p-values* > 0.05). No relation was found between force measures and the total MDS-UPDRS-III and MDS-UPDRS-III subscores (*p-values* > 0.05).

## Discussion

In the present study, we examined force control during an upper and a lower limb force production task in individuals with early-stage PD and a group of age- and gender-matched healthy older adults. We present four novel observations. First, early-stage PD were slower at relaxing force during the pinch grip task relative to healthy individuals, and those PD who were slower at releasing the force were also the PD who presented with more rhythm interruptions during a rapid alternating movement. Second, performance on the lower limb task was also impaired in PD. Specifically, PD showed a slower rate of force contraction and a slower rate of force relaxation during the ankle dorsiflexion task compared to controls. Third, the performance on the lower limb task correlated with disease severity as assessed by the Hoehn and Yahr stage, suggesting that speed-related force control deficits in the lower limb may be more severe in the more advanced stages of the disease. Fourth, force variability measures for the two tasks did not differ between groups, but force accuracy was reduced during the foot task compared to the hand task in both PD and controls.

Consistent with previous work, the performance of the PD group on the upper limb task was characterized by a slower rate of force relaxation [37, 40, 42, 52]. Adequate and well-timed muscle relaxation is key to the optimal performance of motor behaviors, and muscle relaxation deficits in the upper limb have been documented extensively in PD but also in other movement disorders affecting the basal ganglia nuclei such as focal dystonia [67, 68]. In the present study, early-stage PD (Hoehn and Yahr stages 1–2) showed not only a slower rate of force relaxation during the hand task but also a longer duration of the force trials, which is in line with prior investigations [37]. Additional findings from this study come to extend the PD motor control literature by showing that these deficits in force relaxation in PD are related to specific components of upper limb bradykinesia. Bradykinesia, the cardinal motor manifestation that needs to be present to confirm a PD diagnosis, is characterized by slowness of movement, associated with a decrement in amplitude or speed, and with hesitations as the movement is continued. It is commonly assessed with items from the motor section of the MDS-UPDRS for which a combination of movement parameters (speed, amplitude, rhythm) are simultaneously rated on a 5-point scale (0 = Normal, 4 = Severe) [62]. The contribution of the three movement parameters to the rating of a movement is rather ambiguous. In this cohort of PD, we complemented the assessment of motor severity with the modified MDS-UPDRS-III protocol available with the Kinesia ONE system (Great Lakes NeuroTechnologies Inc, Cleveland, OH). The wearable motion sensor allowed us to obtain a more objective assessment of motor symptoms in PD, and a breakdown of the upper limb bradykinesia score into a rating of the speed, amplitude, and rhythm of the movements (Table 1). A recent review paper of mild parkinsonian signs suggested that mild motor changes in the elderly population in the form of a slightly higher MDS-UPDRS-III score in the absence of comorbidities could be an indicator of an increased risk for PD [69]. The mean total MDS-UPDRS-III score for our control cohort was 2.29 points. While the score is not alarmingly high for an older population, we cannot rule out the presence of mild parkinsonian symptoms. Therefore, we advocate the use of more objective measures that could complement existing clinical scales in evaluation of motor changes not only in PD but also in healthy aging.

Importantly, in this study we found that PD who had a slower rate of decrease during the pinch grip task, scored higher on the rhythm component of a more complex movement (i.e., pronation-supination/wrist rotation). This finding begins to elucidate the specific impairments in neuromuscular control mechanisms (e.g., relaxation processes) that underlie poor performance on clinically assessed movements. In this study, we also ran correlation analyses in PD between speed-related measures and the severity of abnormalities in the speed, amplitude and rhythm of the finger tapping and hand grasping movements as assessed by the Kinesia ONE device, and surprisingly, none of them were significant. The presence or the lack of a correlation would have to be determined in future studies that employ both a large and more diverse cohort of PD. Most of the patients recruited in this study had a Hoehn and Yahr score of 1 and 2 (see Table 3) and it could be that the limited spread of the clinical ratings corresponding to features like speed and amplitude masked a relation between clinical and force variables.

**Table 3:** 

Variables	H & Y Stage 1	H & Y Stage ≥2
N	5 (H&Y-1 = 5)	15 (H&Y-2 = 14 | H&Y-3 = 1)
Age (Years)	71.20 (8.93)	66.00 (8.64)
Sex (Male | Female)	2 | 3	9 | 6
Handedness (Right | Left)	5| 0	13 |2
Tested Side (Right | Left)	2 |3	5 | 10
Tested Side (Dominant | Non-Dominant)	2 | 3	7 |8
MVC–Tested Hand	36.00 (11.02)	54.33 (20.13)
MVC–Tested Foot	24.00 (11.47)	64.07 (42.56)
MoCA	25.40 (0.89)	27.47 (2.39)
Disease Duration (Months)	37.00 (34.14)	63.47 (45.94)
Total LEDD	332.00 (321.12)	596.53 (650.48)
MDS-UPDRS-III—Total Score	27.00 (9.03)	32.60 (11.22)
MDS-UPDRS-III—Tested Hand	17.40 (15.47)	18.87 (20.25)
MDS-UPDRS-III—Tested Foot	5.00 (5.29)	5.40 (3.48)
MDS-UPDRS-III—Bradykinesia Tested Hand	8.40 (1.95)	9.07 (4.74)
MDS-UPDRS-III—Bradykinesia Tested Foot	2.80 (1.30)	3.60 (1.60)

The table lists the sociodemographics, clinical characteristics and several behavioral measures for the PD patients group by the Hoehn and Yahr stage. Data are count or mean ± 1 standard deviation (SD). Abbreviations: HY = Hoehn and Yahr Staging Scale; LEDD = levodopa equivalent daily dose; MoCA = Montreal Cognitive Assessment; MDS-UPDRS-III = the motor section of the Movement Disorder Society Unified Parkinson’s Disease Rating Scale; mg = milligram; MVC = maximum voluntary contraction; N = Newtons.

With respect to the rate of force development, although we observed a trend indicating a lower rate of force increase during the hand task in PD relative to controls, this group difference was small and did not reach significance. Previous studies found differences in force development in PD across a variety of upper limb tasks [36, 37, 42, 44, 51–55]. Discrepancies here could be due to methodological differences as well as cohort characteristics. For instance, many of these previous studies provide the total MDS-UPDRS-III (or UPDRS-III) as a measure of disease severity. However, the total score is a crude measure of motor disability and the contribution of the four cardinal motor symptoms and severity of different limbs to this score is unclear. Differences in the degree of impairment in force generation may therefore be related to the severity of the symptoms in the tested limb. Moreover, differences in the topography of dopaminergic depletion within the striatum as well as differences in force demands could be plausible explanations. It is well known that neurons of the basal ganglia circuit play an important role in promoting or suppressing movements, acting like the “gas” and “brake” pedals of the motor circuitry [70–72]. It could well be that in this PD cohort, the pathophysiology of the basal ganglia affects the indirect, inhibitory pathway to a larger extent. Moreover, the required submaximal contraction at 15% of MVC may be less challenging when produced with the hand than the foot.

Our results indicated that force control deficits in early-stage PD are present also in the lower limb, thus indicating an impaired capacity in PD to produce submaximal and rapid force across multiple effectors. Thus far, numerous force control studies in PD placed an emphasis on studying upper limb function, in part motivated by the prevalence of upper extremity symptoms in the PD population [8, 25, 73]. Force control studies of lower extremities in PD are scarce. A key study assessing muscles important for locomotion found that PD had weaker hip flexors, ankle plantar flexors and dorsiflexors compared to age-matched controls and greater force variability across the lower extremity [50]. Most recently force control deficits in the lower extremity were assessed in PD subtypes (tremor-dominant–TD; postural instability and gait difficulty–PIGD), using a knee extension task that showed that PD had lower peak forces and rates of force development compared to controls, regardless of the subtype [74]. Of note, previous investigations focused on either variability or speed measures. To our knowledge, this is the first investigation to concurrently evaluate force control deficits in in the upper and lower extremity in PD using a robust isometric force control paradigm commonly used in PD [37], as well as both speed- and variability-based force characteristics. A novel finding in our study was that PD were slower at contracting and relaxing force during ankle dorsiflexion than controls. The results point to a great need for more objective assessments of lower limb symptoms in PD, assessment that could be sensitive at detecting disease effects even in the early stages of the disease. The rate control deficits reported in this study seem to appear in the context of minimal lower limb impairment as determined by the MDS-UPDRS-III (i.e., a higher score in PD for the hand items than for the foot items; Table 1). The latter is likely due to the fact that clinical scales such as MDS-UPDRS focus for the most part on upper limb function. Currently, only two items are used to assess the severity of bradykinesia in the lower extremity (items 3.7—toe tapping and 3.8—leg agility). Interestingly, the force analysis revealed no limb effect for the rate of force development and force relaxation in PD. An additional interesting finding in our study is the relation between speed-related force measures and the severity of symptoms as assessed by the Hoehn and Yahr stage. Specifically, we found that the rate of force contraction and force relaxation were more impaired in the more advanced stages of the disease (Hoehn and Yahr stage 2 > Hoehn and Yahr stage 1). While this result emphasizes the need for designing lower limb interventions tailored to the early stages of the disease, interventions that could have long-term consequences such as the reduction in the risk of falling which is typically increased in PD [75, 76], it is important to acknowledge the limitations of this analysis such as small sample size (particularly for Hoehn and Yahr stage 1) and limited power. Larger studies sampling the various stages of PD as well as longitudinal studies will be critical to a better understanding of the effect of disease progression on force control deficits.

Lastly, another important parameter of movement performance in addition to speed-related parameters, is force variability. In this study, various measures of force variability were quantified to determine if PD’s force control is impaired. Interestingly, the results of the force analysis indicated group differences in speed-related measures but not variability-related measures. Force variability was similar across groups and as expected, was greater in the foot task than in the hand task. Similar results were found in a small group of young adults who performed upper and lower limb rapid movements using different postures [77]. The standard deviation of the peak force was greater for the lower limb than for the upper limb. Here, the task demand of producing a low-force contraction with the lower extremity, which typically has a greater maximal strength than the upper limb, could potentially explain the higher variability during the foot task. Larger muscles of the lower extremity have higher innervation numbers than small muscles in the hand and this anatomical feature of the lower extremity favors power over precision [78]. It is also possible that the upper limb might have better control of force (e.g., force steadiness) than the lower limb because of its extensive use in daily activity, especially fine motor tasks. Moreover, it is well known that movements requiring precise control of our hands use larger amounts of neural tissue, with a significant portion of the primary motor cortex devoted to finger and hand movements, and a smaller portion devoted to leg movements [79]. Future studies should focus on studying the characteristics of force production (in particular, in the lower extremity) under different conditions employing greater percentages of the MVC.

Together, these results demonstrate an abnormal isometric force profile not only in the upper limb but also in the lower limb in early-stage PD. Specifically, the force profile is abnormal in terms of rate of force development and relaxation and rate control deficits in the ankle in PD seem to become more severe with disease progression. Equally important is the observation that the rate of force control relaxation but not force development explains some of the variance in impaired rhythmic control of the hand in PD. Finally, results also point to the need for intervention studies focused on improving force control in the lower extremity in PD, which in turn could lead to improvements in the stability of balance during standing and walking, which are known to be impaired in PD.

## Supporting information

S1 Data (XLSX)
